# Comparison of Low-Density Lipoprotein Cholesterol (LDL-C) Goal Achievement and Lipid-Lowering Therapy in the Patients With Coronary Artery Disease With Different Renal Functions

**DOI:** 10.3389/fcvm.2022.859567

**Published:** 2022-05-10

**Authors:** Shuang Zhang, Zhi-Fan Li, Hui-Wei Shi, Wen-Jia Zhang, Yong-Gang Sui, Jian-Jun Li, Ke-Fei Dou, Jie Qian, Na-Qiong Wu

**Affiliations:** Cardiometabolic Center, National Center for Cardiovascular Diseases, Fuwai Hospital, Chinese Academy of Medical Science & Peking Union Medical College, Beijing, China

**Keywords:** coronary artery disease, renal function, LDL-C, goal achievement, lipid-lowing therapy

## Abstract

**Aim:**

The aim of this study was to evaluate the relationship between renal function and low-density lipoprotein cholesterol (LDL-C) goal achievement and compare the strategy of lipid-lowering therapy (LLT) among the patients with coronary artery disease (CAD) with different renal functions.

**Methods:**

In this study, we enrolled 933 Chinese patients with CAD from September 2020 to June 2021 admitted to the Cardiometabolic Center of Fuwai Hospital in Beijing consecutively. All individuals were divided into two groups based on their estimated glomerular filtration rate (eGFR). The multiple logistical regression analysis was performed to identify and compare the independent factors which impacted LDL-C goal achievement in the two groups after at least 3 months of treatment.

**Results:**

There were 808 subjects with eGFR ≥ 60 ml/min/1.73 m^2^ who were divided into Group 1 (G1). A total of 125 patients with eGFR <60 ml/min/1.73 m^2^ were divided into Group 2 (G2). The rate of LDL-C goal attainment (LDL-C <1.4 mmol/L) was significantly lower in G2 when compared with that in G1 (24.00% vs. 35.52%, *P* = 0.02), even though there was no significant difference in the aspect of LLT between the two groups (high-intensity LLT: 82.50% vs. 85.60% *P* = 0.40). Notably, in G1, the proportion of LDL-C goal achievement increased with the intensity of LLT (23.36% vs. 39.60% vs. 64.52% in the subgroup under low-/moderate-intensity LLT, or high-intensity LLT without proprotein convertase subtilisin/kexin type 9 (PCSK9) inhibitor (PCSK9i), or high-intensity LLT with PCSK9i, respectively, *P* < 0.005). In addition, in G2, there was a trend that the rate of LDL-C goal achievement was higher in the subgroup under high-intensity LLT (26.60% in the subgroup under high-intensity LLT without PCSK9i and 25.00% in the subgroup under high-intensity LLT with PCSK9i) than that under low-/moderate-intensity LLT (15.38%, *P* = 0.49). Importantly, after multiple regression analysis, we found that eGFR <60 ml/min/1.73 m^2^ [odds ratio (OR) 1.81; 95%CI, 1.15–2.87; *P* = 0.01] was an independent risk factor to impact LDL-C goal achievement. However, the combination strategy of LLT was a protective factor for LDL-C goal achievement independently (statin combined with ezetimibe: OR 0.42; 95%CI 0.30–0.60; *P* < 0.001; statin combined with PCSK9i: OR 0.15; 95%CI 0.07–0.32; *P* < 0.001, respectively).

**Conclusion:**

Impaired renal function (eGFR <60 ml/min/1.73 m^2^) was an independent risk factor for LDL-C goal achievement in the patients with CAD. High-intensity LLT with PCSK9i could improve the rate of LDL-C goal achievement significantly. It should be suggested to increase the proportion of high-intensity LLT with PCSK9i for patients with CAD, especially those with impaired renal function.

## Introduction

In the past decades, the global prevalence of arteriosclerotic cardiovascular disease (ASCVD) has increased considerably; especially, coronary artery disease (CAD) is the leading cause of mortality and morbidity worldwide. Lipid-lowering therapy (LLT) plays an important role in the treatment of CAD by reducing plasma low-density lipoprotein cholesterol (LDL-C) levels (1). Statin is the cornerstone of LLT. Furthermore, ezetimibe and proprotein convertase subtilisin/kexin type 9 (PCSK9) inhibitors (PCSK9i) are important non-statin lipid-lowering choices.

As the chronic disease, chronic kidney disease (CKD) is a gradual loss of renal function over years and is characterized by an estimated glomerular filtration rate (eGFR) of <60 ml/min/1.73 m^2^ (1, 2). The burden of CKD is substantial worldwide. More than 10% of the population were affected by the CKD and the death rate caused by CKD will increase to get 14 per 100,000 people by 2030 (2). CKD has close ties with chronic inflammation, increased oxidative stress and dyslipidemia, and these abnormalities elevate the cardiovascular risk (2, 3). With the eGFR declines and kidney disease progresses from CKD stage Group 1 (G1) to stage Group 5 (G5), the proportion of patients with CKD who die from the cardiovascular disease will increase. Atherosclerotic cardiovascular events represent the most cause of death in patients with CKD (2–5). Renal dysfunction is closely related to dyslipidemia (1, 4). Dyslipidemia in CKD is characterized by hypertriglyceridemia and low high-density lipoprotein (HDL), which is major risk factors for CAD. In the early stages of renal dysfunction, patients with CKD may develop dyslipidemia. Dyslipidemia progresses with deterioration of renal function (4). Unfortunately, previous studies have showed that the rate of LDL-C goal achievement (LDL-C <1.8 mmol/L) was low in the patients with ASCVD (1). Moreover, the latest ESC/EAS guideline recommended that all patients with ASCVD should control their LDL-C level more strictly, lower than 1.4 mmol/L, and reduction ≥ 50% from baseline (6). Currently, there were limited studies focusing on the relationship between the renal function and the rate of LDL-C goal achievement in patients with CAD at the era of PCSK9 inhibitors. The aim of our study was to identify if the renal function is the independent risk factor to impact the selection of high-intensity LLT (especially high-intensity LLT with PCSK9i)or LDL-C goal achievement in real clinical practice.

## Methods

### Study Design and Patient Selection

This study was a prospective, observational cohort study. We enrolled 933 patients with CAD consecutively in the Cardiometabolic Center of Fuwai Hospital (Beijing, China) from September 2020 and June 2021. The inclusion criteria included: 1) coronary angiography showing at least one coronary artery (left anterior descending artery, left circumflex artery, or right coronary artery) stenosis ≥50% and 2) detailed medical records and laboratory data. The exclusion criteria included: 1) severe hepatic dysfunction (aspartate aminotransferase [AST] or/and alanine aminotransferase [ALT] > 3 times the upper limit of normal, 2) life expectancy <3 months, 3) severe blood system disease, systematic inflammatory disease, and malignant disease, 4) contraindication to any LLT, 5) severe renal dysfunction, including CKD Stages 4–5 or dialysis, and 6) receiving some medicines, such as cyclosporine treatment for CKD, which could influence serum concentration of lipid-lowering medicines.

All participants were divided into two groups, and the patients with eGFR ≥ 60 ml/min/1.73 m^2^ were in Group 1 (G1) and those with eGFR <60 ml/min/1.73 m^2^ were in Group 2 (G2). Hypertension was diagnosed by systolic blood pressure (SBP) ≥ 140 mmHg and/or diastolic blood pressure (DBP) ≥ 90 mmHg or receiving antihypertensive therapy. If the fasting plasma glucose was at least 7.0 mmol/L or the patient's 2-h plasma glucose from the oral glucose tolerance test was at least 11.1 mmol/L, or those receiving hypoglycemic treatment, the patients were considered diabetes mellitus (DM). The high-intensity LLT was defined as high-intensity statins (rosuvastatin 20 mg per day or atorvastatin 40–80 mg per day), any-dose statin plus ezetimibe, any-dose statin plus PCSK9i with (or without) ezetimibe, and ezetimibe with PCSK9i or PCSK9i monotherapy, while low-/moderate-intensity LLT was defined as low/moderate statin monotherapy or ezetimibe monotherapy.

### Laboratory Tests

All patients' plasma samples were collected in the morning after overnight fasting. Plasma levels of lipid profile (e.g., total cholesterol [TC], LDL-C, triglyceride [TG], HDL-C, apoA, and apoB) were measured by an automatic biochemistry analyzer (Hitachi 7150, Tokyo, Japan). The serum Lp(a) levels were measured through an immune-turbidimetry assay (LASAY Lp(a) auto; SHIMA Laboratories, Tokyo, Japan).

### Follow-Up

The patients were followed up in 3 months of intervals through telephone or clinical interview. The discontinuation of LLT means that the patients did not take lipid-lowering medicine for >30 days.

### Statistical Analysis

The Kolmogorov–Smirnov test was used to test the distribution pattern. Continuous variables (mean ± SD) and medians with interquartile ranges between the two groups were compared using the unpaired Student's *t*-test or the Mann–Whitney *U* test. Categorical variables (frequencies) were compared using chi-square statistics or the Fisher exact test. The univariate and multiple logistic regression models were constructed to calculate adjusted odds ratios (OR) and 95% confidence intervals (CIs) for the factors that impact LDL-C target achievement. All statistical testing was 2-sided at a significance level of 0.05. Analyses were performed using the R language statistical software (version 4.0.4, Feather Spray; The R Foundation for Statistical Computing, Vienna, Austria).

## Results

### Patient Characteristics

The demographic and clinical characteristics of 933 patients are presented in Table 1. In G1, there were 808 patients with eGFR ≥ 60 ml/min/1.73 m^2^. In addition, 125 patients with eGFR <60 ml/min/1.73 m^2^ were divided into G2. In the whole participants, the average age was 58.4 ± 10.1 years, 74.91% of patients were men, and the mean eGFR was 76.0 (65.0–86.0) ml/min/1.73 m^2^. However, the patients in G2 were older (63.45 ± 8.84 vs. 57.6 ± 9.99 years, *P* < 0.0001) and there were only few male patients in G2 (58.4% vs. 77.48%, *P* < 0.0001). In G2, there tended to be more comorbidities, such as hypertension (76.0% vs. 57.67%, *P* < 0.0001), DM (40.8% vs. 27.72%, *P* = 0.004), and peripheral vascular disease (6.40% vs. 2.48%, *P* = 0.03). Current smoking was prevalent in G1 (41.34% vs. 28.0%, *P* = 0.01). As for the lipid profiles, TG was significantly higher in G2 [1.66 (1.19–2.30) mmol/L vs. 1.45 (1.05–2.06) mmol/L, *P* = 0.01]. There were no significant differences in TC, HDL-C, and LDL-C levels in both the groups.

**Table 1:** The baseline characteristics of total participants including G1 and G2.

**Characteristics**	**Total (*n* = 933)**	**G1 (*n* = 808)**	**G2 (*n* = 125)**	***P*-value**
Male	699 (74.91)	626 (77.48)	73 (58.40)	<0.0001
Age	58.4 ± 10.10	57.60 ± 9.99	63.45 ± 8.84	<0.0001
BMI,kg/m^2^	26.06 ± 3.15	25.99 ± 3.15	26.49 ± 3.09	0.10
SBP,mm Hg	136.91 ± 17.19	136.79 ± 17.09	137.68 ± 17.93	0.59
DBP,mm Hg	78.87 ± 10.54	79.01 ± 10.57	77.95 ± 10.34	0.30
History of PCI	256 (27.43)	215 (26.61)	41 (32.80)	0.18
History of CABG	11 (1.17)	8 (0.99)	3 (2.40)	0.36
History of MI	106 (11.36)	87 (10.77)	19 (15.20)	0.19
PAD	28 (3.0)	20 (2.48)	8 (6.40)	0.03
Stroke	55 (5.89)	50 (6.19)	5 (4.00)	0.45
Hypertension	561 (60.12)	466 (57.67)	95 (76.00)	<0.0001
Hyperlipidemia	631 (67.63)	537 (66.46)	94 (75.20)	0.07
DM	275 (29.47)	224 (27.72)	51 (40.80)	0.004
Current smoking	369 (39.55)	334 (41.34)	35 (28.00)	0.01
History of CAD	95 (10.18)	85 (10.52)	10 (8.00)	0.48
LVEF	62.09 ± 5.84	62.27 ± 5.41	60.92 ± 8.03	0.02
GLU,mmol/L	7.03 ± 2.72	6.90 ± 2.53	7.89 ± 3.63	0.0001
Scr,umol/L	86.76 ± 19.97	82.89 ± 13.19	111.69 ± 33.74	<0.0001
Bun,mmol/L	5.89 ± 1.55	5.74 ± 1.47	6.84 ± 1.69	<0.0001
TG, mmol/L	1.48 (1.07–2.08)	1.45 (1.05–2.06)	1.66 (1.19–2.30)	0.01
TC, mmol/L	4.05 ± 1.07	4.03 ± 1.06	4.15 ± 1.11	0.26
HDL, mmol/L	1.15 ± 0.31	1.15 ± 0.31	1.16 ± 0.34	0.72
LDL, mmol/L	2.31 ± 0.88	2.30 ± 0.87	2.39 ± 0.95	0.26
LDL <1.4mmol/L	108 (11.58)	94 (11.63)	14 (11.20)	1.00
LDL <1.8mmol/L	274 (29.36)	243 (30.07)	31 (24.80)	0.27
eGFR,ml/min/1.73m^2^	76.0 (65.0–86.0)	94.2 (75.9–112.49)	54.13 (53.11–55.15)	<0.0001
Lp (a),mg/L	183.99 (77.10–399.10)	190.17 (78.36–403.54)	164.52 (70.26–397.24)	0.49
hs-crp,mg/L	2.01 ± 2.64	1.97 ± 2.62	2.30 ± 2.81	0.20
apoA,g/L	1.23 ± 0.24	1.22 ± 0.24	1.26 ± 0.23	0.15
apoB,g/L	0.73 (0.59–0.89)	0.73 (0.58–0.88)	0.74 (0.62–0.90)	0.37
nt-proBNP	207.22 ± 524.83	180.60 ± 425.06	378.42 ± 925.84	0.0001
HbA1C,%	6.59 ± 1.65	6.53 ± 1.61	7.05 ± 1.84	0.0011
Medications at baseline				
Statins	667 (71.49)	570 (70.54)	97 (77.60)	0.13
Aspirin	671 (71.92)	579 (71.66)	92 (73.60)	0.73
Clopidogrel	301 (32.26)	257 (31.81)	44 (35.20)	0.51
β-blockers	381 (40.84)	330 (40.84)	51 (40.80)	1
Nitrate	325 (34.83)	278 (34.41)	47 (37.60)	0.55
Calcium channel blockers	159 (17.04)	125 (15.47)	34 (27.20)	0
ACEI/ARB	193 (20.69)	161 (19.93)	32 (25.60)	0.18
Diuretic	10 (1.07)	8 (0.99)	2 (1.60)	0.88

*BMI, body mass index; CABG, coronary artery bypass graft; PCI, percutaneous coronary intervention; MI, myocardial infarction; SBP, systolic blood pressure; DBP, diastolic blood pressure; DM, diabetes mellitus; LVEF, left ventricular ejection fraction; eGFR estimated glomerular filtration rate; TC, total cholesterol; TG, triglyceride; HDL-C, high-density lipoprotein; LDL-C, low-density lipoprotein; hs-CRP, high-sensitivity C-reactive protein; Scr, serum creatinine; Bun, blood urea nitrogen; apoA; NT-proBNP, N-terminal pro-brain natriuretic peptide; Lp(a), lipoprotein(a); Continuous variables are presented as mean ± SD or medians with interquartile ranges. Categorical variables are presented as proportions*.

### Strategy of LLT at the Baseline and Follow-Up Period

**Figure 1:**
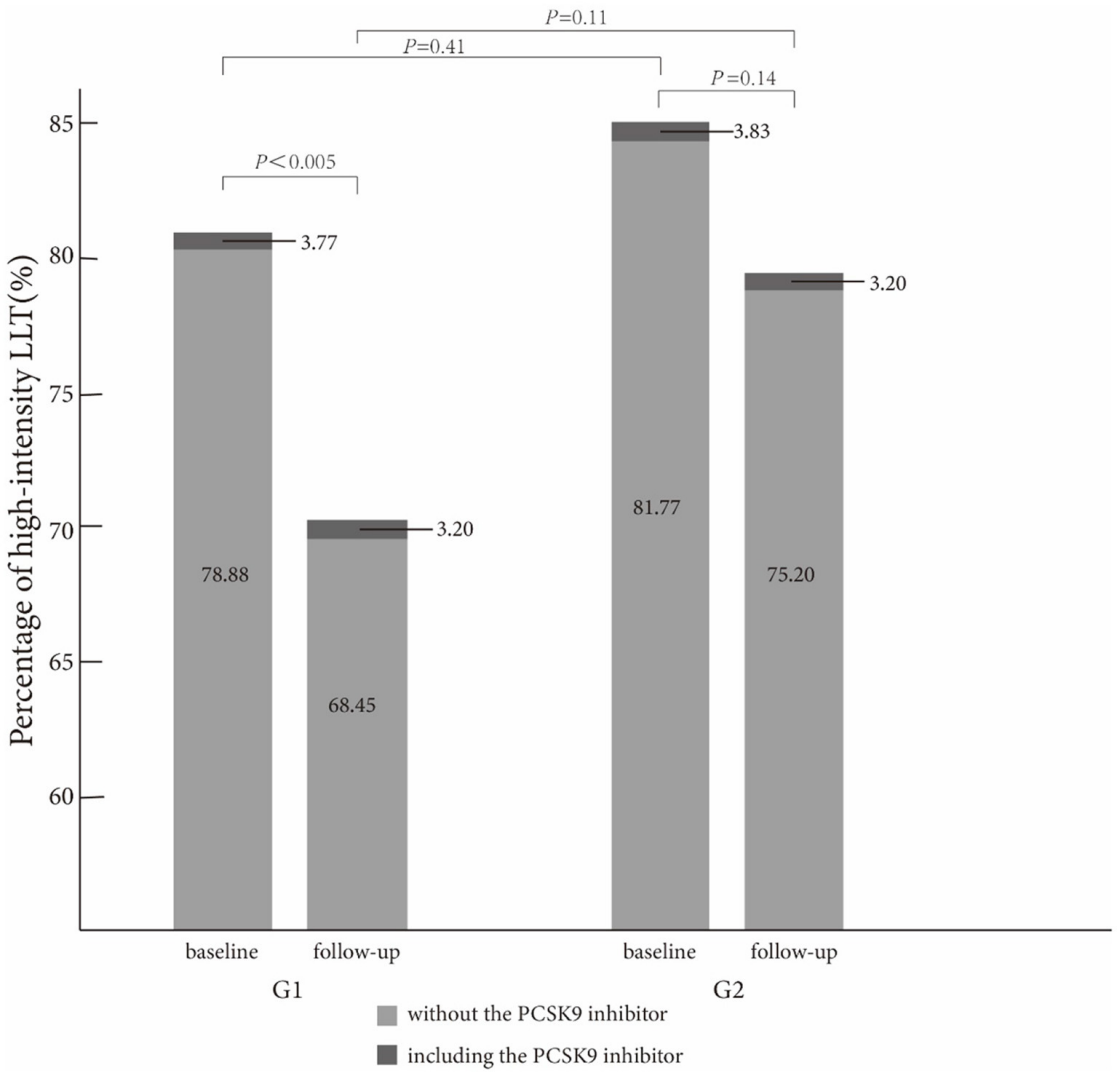
Comparison of high-intensity lipid-lowering therapy strategies between the two groups at baseline and follow-up.

**Figure 2:**
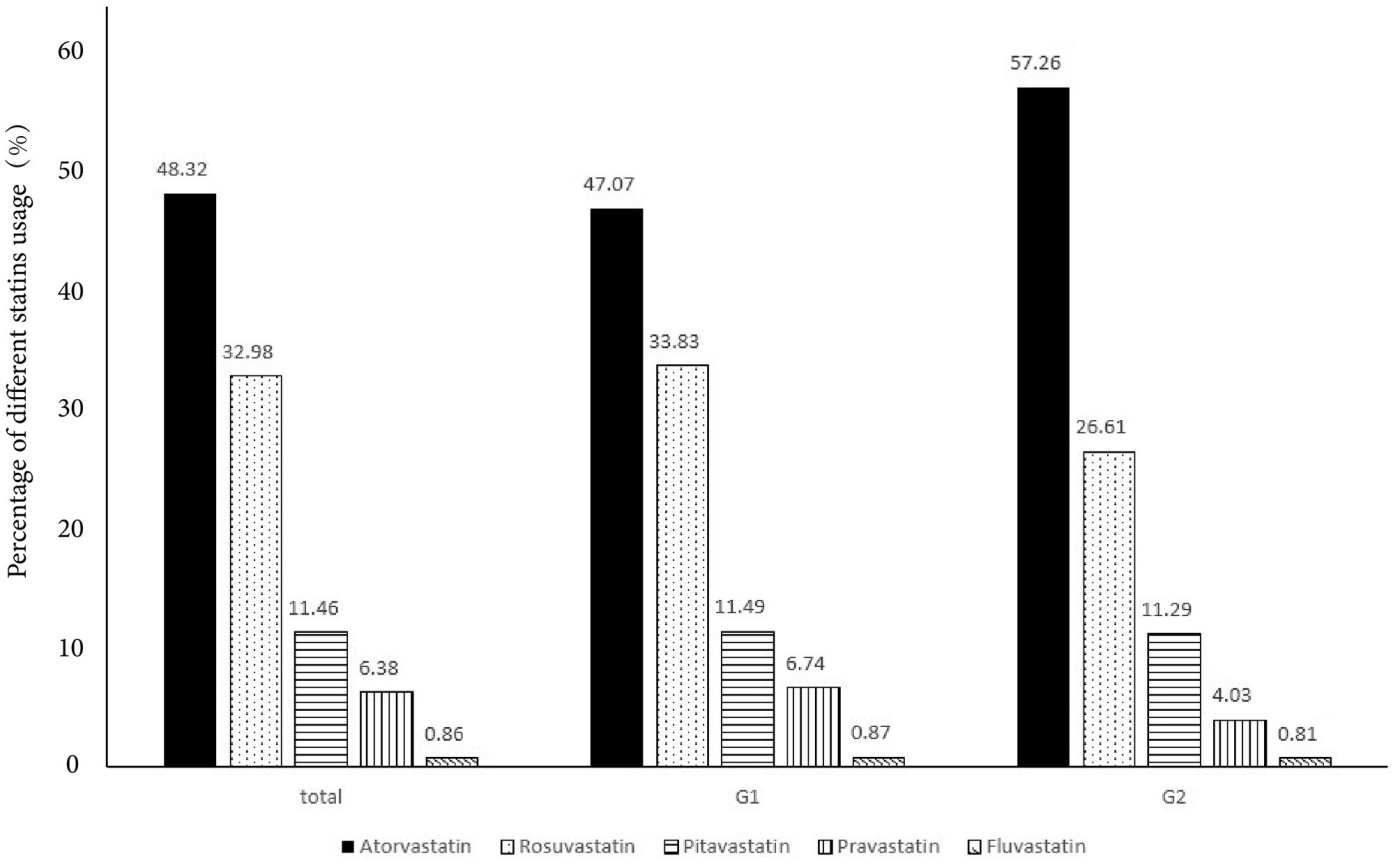
The percentage of different statins used in total and different groups.

In Table 2, high-intensity LLT (low-/moderate-intensity statin + ezetimibe) was the most common strategy at baseline (78.09% in G1 vs. 80.06% in G2) and during the follow-up (66.71% in G1 vs. 74.40% in G2) period. There was no significant difference in strategies of LLT between the two groups at baseline (*P* = 0.41, Figure 1) and at follow-up period (*P* = 0.11, Figure 1). In addition, PCSK9i application was relatively low in both groups at baseline (3.77% in G1 vs. 3.20% in G2) and during the follow-up period (3.83% in G1 vs. 3.2% in G2). Moreover, at baseline, atorvastatin was the most common prescription in statins (48.32%), followed by rosuvastatin (32.98%) (Figure 2). Very low proportions (1.07%) of subjects were under LLT with high-intensity statin monotherapy.

**Table 2:** Comparison of the lipid-lowering therapy (LLT) strategies between the two groups at baseline and follow-up period.

	**Total (*n* = 933)**	**G1 (*n* = 808)**	**G2 (*n* = 125)**
**Baseline**, ***n*** **(%)**			
Low/moderate intensity LLT			
Low/moderate intensity statin alone	149 (15.97)	132 (16.34)	17 (13.60)
Ezetimibe alone	8 (0.87)	7 (0.87)	1 (0.80)
High intensity LLT			
Low/moderate intensity statin+Ezetimibe	733 (78.56)	631 (78.09)	102 (81.60)
Moderate intensity statin+Ezetimibe+PCSK9i	27 (2.89)	25 (3.09)	2 (1.60)
High intensity statin+Ezetimibe	7 (0.75)	6 (0.74)	1 (0.80)
Moderate intensity statin+PCSK9i	4 (0.43)	2 (0.25)	2 (1.60)
High intensity statin+Ezetimibe+PCSK9i	2 (0.21)	2 (0.25)	0 (0.00)
High intensity statin	1 (0.16)	1 (0.18)	0 (0.00)
Ezetimibe+PCSK9i	1 (0.16)	1 (0.18)	0 (0.00)
**Follow-up**, ***n*** **(%)**			
Low/moderate intensity LLT			
Low/moderate intensity statin alone	225 (24.12)	200 (24.75)	25 (20.00)
Ezetimibe alone	15 (1.61)	14 (1.73)	1 (0.80)
High intensity LLT			
Low/moderate intensity statin+Ezetimibe	632 (67.74)	539 (66.71)	93 (74.40)
Low/moderate intensity statin+Ezetimibe+PCSK9i	25 (2.68)	23 (2.85)	2 (1.60)
High intensity statin+Ezetimibe	9 (0.96)	8 (0.99)	1 (0.80)
Low/moderate intensity statin+PCSK9i	8 (0.86)	6 (0.74)	2 (1.60)
PCSK9i alone	1 (0.11)	1 (0.12)	0 (0.00)
High intensity statin	1 (0.11)	1 (0.12)	0 (0.00)
High intensity statin+Ezetimibe+PCSK9i	1 (0.11)	1 (0.12)	0 (0.00)
Discontinuation of any LLT, n (%)	16 (1.71)	15 (1.86)	1 (0.80)

*LLT, lipid-lowering therapy; PCSK9i, PCSK9 inhibitor. Continuous variables are presented as mean ± SD or medians with interquartile ranges. Categorical variables are presented as proportions*.

The average LDL-C level during the follow-up period was higher in G2 than in G1 (1.91 ± 0.68 mmol/L in G2 vs. 1.71 ± 0.68 mmol/L in G1, *P* = 0.0022). The percentage of LDL-C target achievement (<1.4 mmol/L) was 35.52% in G1 and 24.00% in G2, respectively (*P* = 0.02, Table 3).

**Table 3:** Comparison of the lipid profile at the follow-up period between the two groups.

	**Total (*n* = 933)**	**G1 (*n* = 808)**	**G2 (*n* = 125)**	***P*-value**
TG, mmol/L	1.19 (0.88–1.70)	1.17 (0.86–1.69)	1.33 (1.03–1.78)	0.01*
TC, mmol/L	3.35 ± 0.95	3.34 ± 0.97	3.47 ± 0.85	0.16
HDL-C, mmol/L	1.14 ± 0.28	1.15 ± 0.28	1.11 ± 0.26	0.17
LDL-C, mmol/L	1.74 ± 0.68	1.71 ± 0.68	1.91 ± 0.68	0.0022*
LDL-C <1.4mmol/L, (%)	317 (33.98)	287 (35.52)	30 (24.00)	0.02*

TC, total cholesterol; TG, triglyceride; HDL-C, high-density lipoprotein; LDL-C, low-density lipoprotein; *indicated the difference between the two groups is statistically significant, P < 0.05. Continuous variables are presented as mean ± SD or medians with interquartile ranges. Categorical variables are presented as proportions.

**Figure 3:**
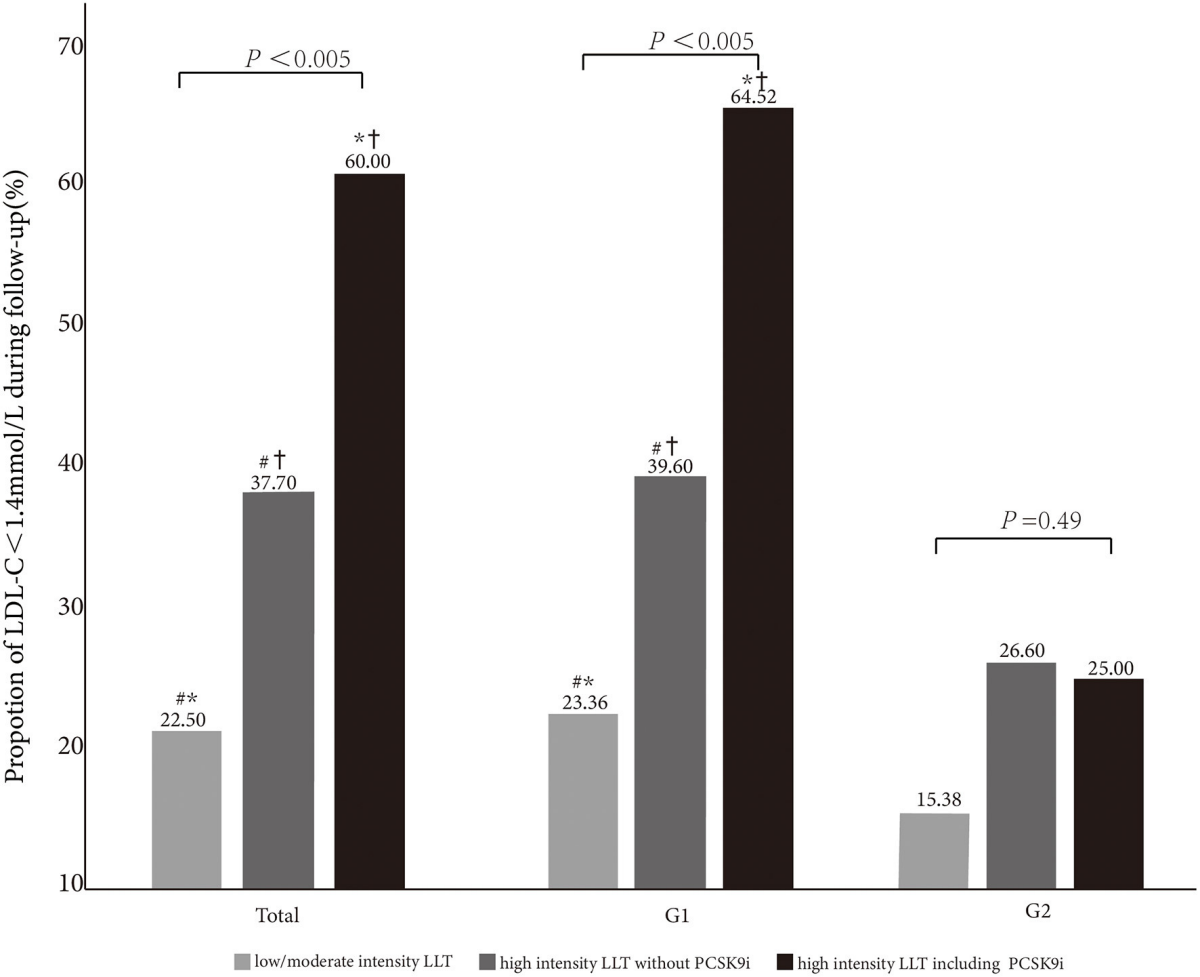
Comparison of LDL-C <1.4 mmol/L goal achievement under different LLT strategies in total and subgroups (G1 and G2). *, #,^†^indicate < 0.05 between the groups.

The proportion of high-intensity LLT strategy had a trend to be higher in G2 than that in G1 during the follow-up period (78.20% without PCSK9i and 3.20% including PCSK9i in G2 vs. 68.45% without PCSK9i and 3.20% including PCSK9i in G1, *P* = 0.11). In addition, the proportion of high-intensity LLT was significantly lower during the follow-up period than that at baseline in G1 (78.88% without PCSK9i and 3.77% including PCSK9i at baseline vs. 68.45% without PCSK9i and 3.20% including PCSK9i during follow-up in G1, *P* < 0.005). However, the proportion of high-intensity LLT did not significantly change in G2 during the follow-up period than that at baseline (81.77% without PCSK9i and 3.83% including PCSK9i at baseline vs. 78.20% without PCSK9i and 3.20% including PCSK9i during follow-up, *P* = 0.14) (Figure 3).

### Attainment of LDL-C Goal During the Follow-Up Period

In Table 3, it summarized the percentage of patients achieving LDL-C target and lipid profiles during the follow-up period. After ≥3 months of LLT treatment, the rate of LDL-C <1.4 mmol/L increased up to 33.99%. However, the rate of LDL-C goal achievement was lower in G2 compared with that in G1 (24.00% vs. 35.52%, *P* = 0.02). The average LDL-C level in G2 was higher than that in G1 during the follow-up period (1.91 ± 0.68 mmol/L vs. 1.71 ± 0.68 mmol/L, *P* = 0.0022).

The patients with the high-intensity LLT are more prone to achieve the LDL-C goal (high-intensity LLT with PCSK9i: 60.0%; high-intensity LLT without PCSK9i: 37.7%; low-/moderate-intensity LLT: 22.50%, *P* < 0.005). In G1, the proportion of LDL-C goal achievement (LDL-C <1.4 mmol/L) increased with the intensity of LLT (23.36% vs. 39.60% vs. 64.52% in the subgroup under low-/moderate-intensity LLT, or high-intensity LLT without PCSK9i, or high-intensity LLT with PCSK9i, respectively, *P* < 0.005). In addition, in G2, there was a trend that the rate of LDL-C goal achievement was higher in the subgroup under high-intensity LLT (26.60% in the subgroup under high-intensity LLT without PCSK9i, 25.00% in the subgroup under high-intensity LLT with PCSK9i) than that under low-/moderate-intensity LLT (15.38%), even though the difference was not statistically significant (*P* = 0.49, Figure 3).

### Factors That Impact LDL-C Goal Achievement

After multivariate logistic regression analysis, previous percutaneous coronary intervention (OR 0.55; 95%CI 0.39–0.77; *P* < 0.001), admission for acute coronary syndrome (ACS) (OR 0.65; 95%CI 0.46–0.91; *P* = 0.01), LLT including statin combination with ezetimibe (OR 0.42; 95%CI 0.3–0.6; *P* < 0.001), and LLT including statin and/or ezetimibe combination with PCSK9i (OR 0.15; 95CI% 0.07–0.32; *P* < 0.001) were all significantly associated with LDL-C goal achievement. In addition, DM (OR 1.50; 95%CI 1.09–2.02; *P* = 0.001), female patients (OR 1.66; 95%CI 1.13–2.44; *P* = 0.009) were significantly associated with LDL-C goal achievement. Importantly, eGFR <60 ml/min/1.73 m^2^ (OR 1.81; 95%CI 1.15–2.87; *P* = 0.01) was also significantly associated with LDL-C goal achievement (Table 4).

**Table 4:** Factors impacting achieving LDL-C <1.4 mmol/L goal for patients treated with LLT.

	**Univariate logistic regression**			**Multivariate logistic regression**		
	**OR**	**95 CI%**	***P*-value**	**OR**	**95 CI%**	***P*-value**
Age	0.85	0.63–1.14	0.28			
Female	1.69	1.21–2.35	0.002*	1.66	1.13–2.44	0.009*
History of PCI	0.67	0.49–0.92	0.01*	0.55	0.39–0.77	<0.001
History of CABG	1.63	0.49–5.38	0.42			
History of MI	1.10	0.72–1.67	0.67			
ACS	0.71	0.52–0.98	0.03*	0.65	0.46–0.91	0.01*
PAD	1.08	0.49–2.37	0.84			
Hypertension	0.84	0.64–1.11	0.22			
DM	0.77	0.57–1.03	0.08	1.50	1.09–2.02	0.01*
smoking	1.31	1.00-1.73	0.05	0.99	0.72–1.36	0.95
eGFR <60 ml/min/1.73 m^2^	1.74	1.23–2.70	0.01*	1.81	1.15–2.87	0.01*
BMI>30	1.00	0.65–1.53	1.00			
lipid-lowering therapy						
Monotherapy (statins)	Ref.			Ref.		
Combination with ezemitibe	0.52	0.37–0.74	<0.001*	0.42	0.30–0.60	<0.001*
Combination with PCSK9i	0.21	0.10–0.44	<0.001*	0.15	0.07–0.32	<0.001*

BMI, body mass index; CABG, coronary artery bypass graft; PCI, percutaneous coronary intervention; MI, myocardial infarction; eGFR estimated glomerular filtration rate; DM, diabetes mellitus; ACS, admission for acute coronary syndrome; *indicated P < 0.05.

## Discussion

In this prospective observational study, we analyzed the factors impacting LDL-C <1.4 mmol/L goal achievement in Chinese patients with CAD and different renal function statuses. Although in the whole participants, the rate of LDL-C <1.4 mmol/L goal achievement reached 33.99% during the follow-up period from 11.57% at baseline; however, the rate of LDL-C <1.4 mmol/L goal achievement was significantly lower in G2 than that in G1 (24.00% vs. 35.52%, *P* = 0.02). At the same time, we found that impaired renal function (eGFR <60 ml/min/1.73 m^2^) was independently associated with LDL-C <1.4 mmol/L goal achievement (OR 1.81; 95%CI 1.15–2.87; *P* = 0.01). From a mendelian randomization, there is a stepwise increased risk of CKD with a higher LDL-C level (hazard ratio [HR] 1.05; 95CI% 0.97-1.13; *P* <0.001) (7). LDL-C and CKD are independent risk factors for cardiovascular events (1). Given the present research results, LDL-C goal achievement might be a challenge for CAD patients with impaired renal functions.

Based on the latest ESC/EAS guideline about the lipid management, LDL-C goal is <1.4 mmol/L and reduction ≥ 50% from the baseline for all the patients with ASCVD (6). Renal function has been well recognized to be associated with CAD and dyslipidemia (1). Concerning to lipid management in the CAD patients with impaired renal function, our findings are consistent with the results of previous studies and highlight a treatment gap between clinical practice and guideline commendation. In the previous study, it had been found that the patients with advanced CKD were less likely to achieve LDL-C target (8, 9). In a prospective cohort study CKD-REIN (NCT03381950), among high-risk patients, 45% of those on statin and/or ezetimibe achieved the LDL-C treatment target (<2.6 mmol/L). Among very high-risk patients, the percentage at goal (<1.8 mmol/L) was 38% for CKD stage G3 and 29% for stage G4/G5. There was a trend toward the higher achievement of LDL-C targets with increasing LLT intensity (adjusted OR for moderate vs. low intensity 1.20; 95%CI 0.92–1.56; high vs. low intensity 1.46; 1.02–2.09; *P* trend = 0.036). In the CKD-REIN study, many patients with CKD stages G3–G5 who were eligible for LLT were not treated, and those on LLT rarely achieved LDL-C targets (8). Kuznik et al. reported that the percentage of LLT increased with CKD stage and the rate of LDL-C <100 mg/dL increased with CKD stage among the patients with below CKD Stage 3b, but the target rate decreased in Stage 4 (10). However, Lin et al. conducted a multi-center study (T-SPARCLE) that enrolled 3,057 individuals and 26.76% of patients with CKD. Those without CKD had a similar equivalent statin potency with the CKD group. Although the result showed that more CKD population achieved the LDL-C goal, there were no statistical significances between the CKD and non-CKD groups (55.75% vs. 54.71%, *P* > 0.05) (11). In the 2001–2010 National Health and Nutritional Examination Survey (NHANES), the use of lipid-lowering agents increased with CKD stage, from 18.1% (Stage 1) to 44.8% (Stage 4). LDL-C goal attainment increased from 35.8% (Stage 1) to 52.8% (Stage 3b) but decreased in Stage 4 (50.7%). From this survey, it was found that individuals with CKD had a high prevalence of CV-related comorbidities. However, attainment of LDL-C goals was low regardless of disease stage (10). In Taiwan CKD care programs conducted by nephrologists-based team from 2006 to 2013, they set 10 goals with treatment target ranges based on the guideline. In this program, they found that the all-goals attainment rate increased from 59.4% at baseline to 60.5% in year 3, with an especially significant improvement for LDL-C (from 46.8% to 67.0%). From the program, they concluded that goal attainment and disease progression were influence by CKD stage. A high goal achievement rate was associated with better preservation of residual renal function (9). Since the previous studies have mainly explored the primary prevention of high-risk patients with CKD, our data concentrated on the second prevention of patients with CAD with various renal functions. From our study, the renal function exerts an adverse impact on the lipid management in patients with CAD. Patients with impaired renal function should be paid more attention.

The proportion of high-intensity LLT strategy had a trend to be higher in G2 than that in G1 during the follow-up period in our study. However, there was no statistically significant difference in the aspect of LLT strategy in both the groups (78.20% without PCSK9i and 3.20% including PCSK9i in G2 vs. 68.45% without PCSK9i and 3.20% including PCSK9i in G1, *P* = 0.11). In addition, we found that the proportion of high-intensity LLT was significantly lower during the follow-up period than that at baseline in G1 (78.88% without PCSK9i and 3.77% including PCSK9i at baseline vs. 68.45% without PCSK9i and 3.20% including PCSK9i during follow-up, *P* <0.005). However, the proportion of high-intensity LLT did not significantly change in G2 during the follow-up period than that at baseline (81.77% without PCSK9i and 3.83% including PCSK9i at baseline vs. 78.20% without PCSK9i and 3.20% including PCSK9i during follow-up, *P* = 0.14). From our study, high-intensity LLT was independently associated with LDL-C goal achievement. High-intensity LLT, such as statin plus ezetimibe, could make it more likely to achieve LDL-C target (OR 0.42; 95%CI 0.3–0.6; *P* <0.001). It was similar that high-intensity LLT, such as statin or ezetimibe combined with PCSK9i, could also achieve LDL-C goal more likely (OR 0.15; 95CI% 0.07–0.32; *P* <0.001). Massy et al. also reported that the combination therapy of LLT recommended by the guidelines could make more patients with CKD to achieve LDL-C goal (8). From the long-term result, Bae et al. reported a median follow-up of 4.2 years, and the combined groups always had the lower LDL-C levels (*P* = 0.025) (12). In this study, the patients under high-intensity LLT were more prone to achieve LDL-C goal, especially under LLT with PCSK9i (22.50% vs. 37.70% vs. 60.00% in the subgroup under low-/moderate-intensity LLT, or high-intensity LLT without PCSK9i, or high-intensity LLT with PCSK9i, respectively, *P* <0.005). It was remarkable that although 68.70% of individuals were under high-intensity LLT with or without PCSK9i, the rate of LDL-C goal achievement (LDL-C <1.4 mmol/L) was only 33.99%. The potential cause was a very low rate of PCSK9i application (only 3.69% and 3.76% at baseline and follow-up, respectively). Similarly, the previous cross-sectional studies have also reported small proportions of PCSK9i in a real-world application (13, 14). From our study, we speculated that high-intensity LLT without PCSK9i was not so enough to achieve a high rate of LDL-C goal achievement. According to the guideline, it is sound to use the PCSK9i after statin combined with ezetimibe (6). PCSK9i application might help more patients to achieve LDL-C <1.4 mmol/L. Actually, our result showed the percentage of PCSK9i kept similar between the baseline and follow-up (baseline 3.69% vs. follow-up 3.76%). Insufficient attention had been paid to the groups with eGFR <60 ml/min/1.73 m^2^ about the lipid management. It might be considerable to prescribe PCSK9i for them more, especially for the patients with impaired renal function.

Except for the effectiveness on lowering LDL-C, LLT also exerts the protective effect on renal function. From the network meta-analysis, statins could lead to a 0.61 (95CI% 0.27–0.95) ml/min/1.73 m^2^ slower annual eGFR decline. When it comes to the efficacy among different statins, there are no substantial differences (15). According to the *post-hoc* analyses from several trials, atorvastatin could improve renal function (16, 17). Statin combination with ezetimibe also had positive effects on renal function (18). The renal safety of ezetimibe had been proven by the Study of Heart and Renal Protection (SHARP) trial, which used simvastatin 20 mg plus ezetimibe 10 mg reduced atherosclerotic events in advanced patients with CKD. Compared with the statins, the combination group was prone to preserve renal function (*P* <0.001) and had less renal events (HR 0.58; 95CI% 0.35–0.95; *P* = 0.032) (19). PCSK9i are currently the most effective lipid-lowering drugs in clinical practice, which could reduce LDL-C level by 50–70% and improve the rate of LDL-C goal achievement significantly (20, 21). The FOURIER trial investigated the influence of evolocumab on the patients with different kidney functions. As for the effect of LDL-C lowering and clinical efficacy and safety of evolocumab, it is consistent across different renal function groups and more effective to reduce the rate of adverse events in the advanced CKD group (20). Alirocumab had a similar effect as evolocumab (21). Based on the current evidence, for the patients with CAD with impaired renal function, it was more difficult to achieve LDL-C goal and it needs to optimize the application of high-intensity LLT, especially improving the proportion of PCSK9i application in order to accomplish a higher rate of LDL-C goal achievement.

Another possible contributing factor for the low rate of LDL-C goal achievement was medication adherence. In our study, LLT strategy was adjusted in 105 patients during the follow-up period when compared with that at discharge and among them, 102 patients switched from statin combination with ezetimibe to moderate-intensity statin monotherapy. In previous studies, the long-term adherence to statins was poor (22). Santoleri et al. showed that an overall adherence rate of ezetimibe was low when compared with statins during the 8-year follow-up period, and a higher percentage of discontinuation of ezetimibe than statins annually (23). In addition to patient self-discontinuation, 4% of patients discontinued ezetimibe because of physicians' suggestion. The physicians' lipid management knowledge had a great influence on the rate of LDL-C goal achievement (24).

There are some limitations in this study. First, it was a single-center observational study; although confounding variables have been statistically excluded, there might be unobserved risk factors. Second, the follow-up period in our study was relatively short; thus, the long-term effect of different renal functions and different LLTs on LDL-C goal achievement in patients with CAD remained uncertain. Third, the sample size was small. Finally, when it comes to the adherence, we did not take a more accurate way to assess the medication using situation except for patients' self-report during the follow-up period.

In summary, in our study, impaired renal function (eGFR <60 ml/min/1.73 m^2^) was an independent risk factor for LDL-C goal achievement in the patients with CAD. High-intensity LLT with PCSK9i could improve the rate of LDL-C goal achievement significantly. It should be suggested to increase the proportion of high-intensity LLT with PCSK9i for patients with CAD, especially those with impaired renal function.

## Data Availability Statement

The raw data supporting the conclusions of this article will be made available by the authors, without undue reservation.

## Ethics Statement

The studies involving human participants were reviewed and approved by Fuwai hospital Ethics Committees. The patients/participants provided their written informed consent to participate in this study. Written informed consent was obtained from the individual(s) for the publication of any potentially identifiable images or data included in this article.

## Author Contributions

NQW: conception/design. YGS, WJZ, JJL, JQ, and KFD: provision of study materials. SZ, HWS, and ZFL: collection and/or assembly of data. SZ: data analysis, interpretation, and manuscript writing. All authors contributed to the article and approved the submitted version.

## Funding

This study was supported by the CAMS Innovation Fund for Medical Sciences (CIFMS) (2021-I2M-1-008).

## Conflict of Interest

The authors declare that the research was conducted in the absence of any commercial or financial relationships that could be construed as a potential conflict of interest.

## Publisher's Note

All claims expressed in this article are solely those of the authors and do not necessarily represent those of their affiliated organizations, or those of the publisher, the editors and the reviewers. Any product that may be evaluated in this article, or claim that may be made by its manufacturer, is not guaranteed or endorsed by the publisher.
